# Regional differences in perceived oral dryness as determined with a newly developed questionnaire, the Regional Oral Dryness Inventory

**DOI:** 10.1007/s00784-020-03276-7

**Published:** 2020-05-07

**Authors:** Zainab Assy, D. H. J. Jager, E. Mashhour, F. J. Bikker, H. S. Brand

**Affiliations:** 1 Department of Oral Biochemistry, Academic Centre for Dentistry Amsterdam, University of Amsterdam and VU University Amsterdam, Room 12N-37, Gustav Mahlerlaan 3004, 1081 LA Amsterdam, The Netherlands; 2 Department of Oral and Maxillofacial Surgery and Oral Pathology, Amsterdam UMC, Amsterdam, The Netherlands; 3 Center for Special Care Dentistry (Stichting Bijzondere Tandheelkunde), Amsterdam, Netherlands

**Keywords:** Dry mouth, Xerostomia, Salivary flow rate, Xerostomia Inventory, Clinical Oral Dryness Score

## Abstract

**Objectives:**

Several questionnaires, such as the internationally validated and frequently used Xerostomia Inventory (XI), have been developed to quantify the subjective feeling of a dry mouth. These questionnaires quantify the overall perception of dry mouth but lack the possibility to differentiate between various intra-oral regions. In this light, a novel questionnaire, the Regional Oral Dryness Inventory (RODI), which quantifies the severity of dryness at various locations in the mouth, was evaluated.

**Materials and methods:**

A retrospective case report study was designed. Data were collected from patients who visited the saliva clinic for Special Care Dentistry in Amsterdam. Data, including the saliva secretion rates, RODI scores, the Xerostomia Inventory (XI) score, and Clinical Oral Dryness Score (CODS), were extracted from the electronic health record system Oase Dental.

**Results:**

A total of 337 patients participated in this study with an average age of 54 ± 17 years. The majority of the patients were female (68.5%). The perceived dryness as determined by the RODI was the highest for the posterior palate and the lowest for the floor of the mouth. The highest correlations were found between the corresponding regions in the RODI and regionally related individual items of the XI and CODS.

**Conclusion:**

There is a significant difference in dry-mouth feeling at different intra-oral locations.

**Clinical relevance:**

Regional evaluation of xerostomia with RODI might improve diagnosis of xerostomia by helping to discriminate between different potential causes of oral dryness in patients and for evaluating the efficacy of mouth-moistening products. RODI is highly accessible and easy to perform in dental practices during routine clinical assessment.

**Electronic supplementary material:**

The online version of this article (10.1007/s00784-020-03276-7) contains supplementary material, which is available to authorized users.

## Introduction

Saliva is a multi-functional fluid, which provides mucosal lubrication and moistening, and protection of the teeth and oral mucosa surface, and plays an important role in digestion, protecting oral tissues, swallowing, taste, and speaking [1, 2]. Therefore, an adequate saliva flow is important for the maintenance of the oral health [3, 4].

Saliva flow can be impaired due to many factors. A reduction in saliva secretion rate can be the result of xerogenic medications, radiotherapy of the head and neck, or systemic diseases such as Sjögren’s syndrome [5–7]. Patients suffering from a reduced salivary flow rate may complain about taste alterations, swallowing difficulties, and a burning sensation in the mouth. Other oral complications include increased risk of ulcerations, caries, gingivitis, periodontitis, and oral *Candida spp.* infections [8, 9].

A reduced salivary flow rate is known as hyposalivation and can objectively be determined by sialometry. Hyposalivation is defined as a salivary flow rate is < 0.1 mL/min at rest or < 0.7 mL/min upon stimulation [8]. In contrast, the subjective sensation of a dry mouth experienced by the patient is called xerostomia [9, 10], which can only be determined with self-reported questionnaires [11–15]. Over the past decades, several questionnaires have been developed to quantify the overall feeling of a dry mouth [11–15]. For example, the Xerostomia Inventory (XI) is an internationally validated and frequently used questionnaire with 11 items on a 5-point Likert scale to quantify the severity of the xerostomia [11].

The sensation of a dry mouth is not solitarily related to the reduction in salivary secretion rate changes but might also be related to the unequal thickness of the saliva film on both soft and hard oral tissue surfaces [16]. To exemplify, the salivary film that remains in the oral cavity after swallowing is the thickest at the dorsal area of the tongue and the thinnest at the hard palate [17–21].

In addition, differences in salivary composition have also been implicated in the perception of dry mouth [19–21]; the salivary mucin MUC5B retains large amounts of water and contributes to the generation of a hydrophilic gel essential for lubrication of the oral epithelium [22–24]. Moreover, MUC5B is the main component that determines the viscoelasticity of saliva [24]. Local variations in the MUC5B concentration have been reported with higher intensity on the hard palate compared with other oral surfaces [18].

In light of these local variations [17, 18, 21], the palate may be more frequently related to xerostomia complaints compared with other areas, e.g., the tongue [19].

So far, xerostomia questionnaires were aimed to quantify the overall feeling of mouth dryness and not the perceived xerostomia at different intra-oral locations. Therefore, the purpose of this study is to evaluate a recently developed questionnaire, Regional Oral Dryness Inventory (RODI), which quantifies the severity of dryness at various locations in the mouth.

## Materials and methods

### Study design

A retrospective case report study was designed. Data were collected from patients older than 18 years, who visited the saliva clinic for Special Care Dentistry in Amsterdam. These patients were referred to the saliva clinic by dentists, general physicians, and medical specialists between January 2014 and April 2019. All the patients included in this study had saliva-related and/or dry-mouth complaints.

The Ethics Review Committee of the Academic Centre for Dentistry Amsterdam (ACTA) confirmed that the Medical Research Involving Human Subjects Act (WMO) does not apply to this study (protocol number 201910). The reporting of this study conforms to the STROBE statement [25].

All the questionnaires and clinical parameters have been collected and interpreted by a single practitioner (DHJJ). A standardized protocol is used for this process, which takes approximately 45 min. All the procedures described in the present study are part of the regular patient care routine in the saliva clinic.

### Data collection methods

The relevant data were extracted by one abstractor (EM) from the electronic health record system Oase Dental (VST Software B.V., Haarlem, The Netherlands). Patients were included when most of the relevant data were present in the record of the patient. The extracted data were registered pseudonymized in a Microsoft Excel under a code number so that the data can no longer be traced back to the patients. The following clinical data were retrieved: gender, age, the Xerostomia Inventory (XI) score, Clinical Oral Dryness Score (CODS), scores on the newly developed Regional Oral Dryness Inventory, and the secretion rates of unstimulated whole saliva (UWS), chewing-stimulated whole saliva (CH-SWS), and citric acid–stimulated whole saliva (A-SWS).

Random checks were done after data entry, by two researchers (EM and ZA), to verify correct transfer of data from the medical record to the case reports. This was performed according to the 100-20 rule in which 100% of the data is checked in 20% of the case reports and 20% of the most important data are checked in 100% of the case reports [26].

### Study variables

#### Subjective oral dryness

Before a patient visited the saliva clinic, he or she received several questionnaires by mail to fill out at home. These questionnaires included the Xerostomia Inventory (XI) which consists of 11 items on a 5-point Likert scale ranging from 1 = “never” to 5 = “very often.” The items are about oral dryness and mouth feel in the patients. Patients indicate in each item how often they suffer from problems with regard to mouth feel and oral dryness. The scores of the 11 items are summed resulting in a total XI score that ranges between 11 (no xerostomia) and 55 (extreme xerostomia) [11].

**Fig. 1:**
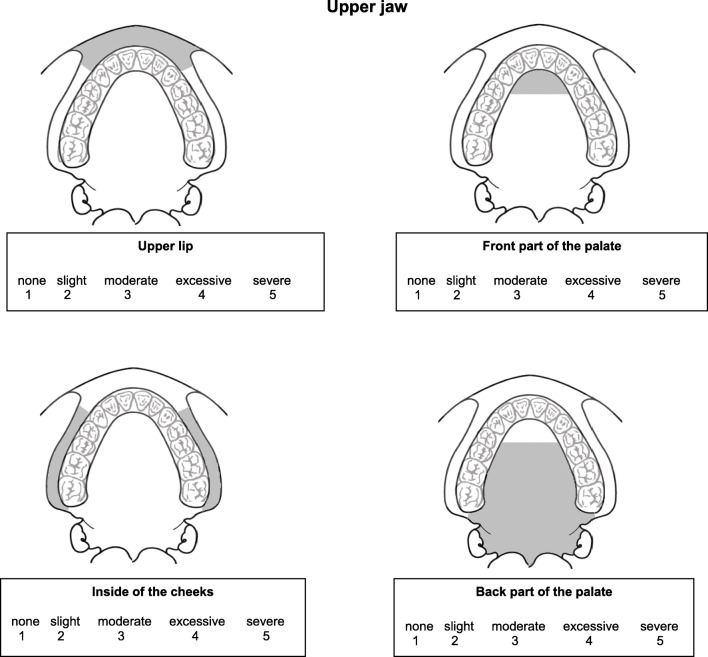

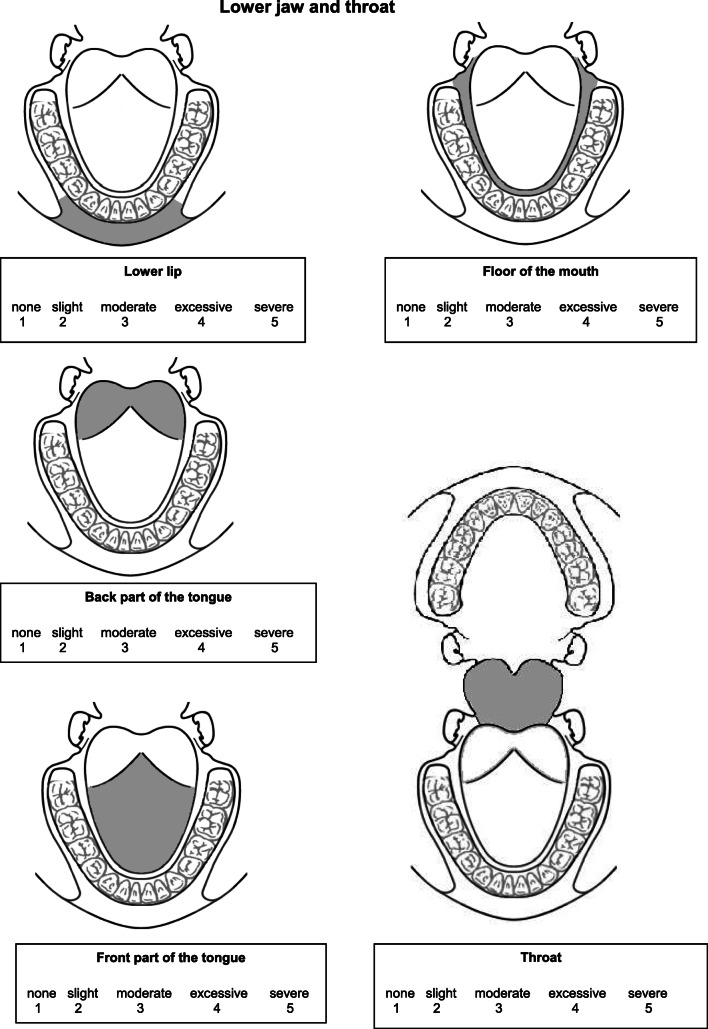
The Regional Oral Dryness Inventory with the nine intra-oral regions and instructions. Regional Oral Dryness Inventory. The following questions are about dryness perception in the mouth during the last 4 weeks. The illustrations below show four different regions in the upper jaw, four different regions in the lower jaw, and an illustration of the throat. Please indicate the severity of dryness for each of these different locations on a scale from 1 to 5, where 1 = no dryness and 5 = severe dryness. It is advisable to answer spontaneously and not spend too much time considering your answer.

In addition, the patients received a newly developed Regional Oral Dryness Inventory (RODI) (see Fig. 1). This questionnaire contains 9 schematic illustrations of different locations in the oral cavity. Four illustrations represent areas in the upper jaw: the upper lip, anterior part of the palate (including the rugae), inside part of the cheeks, and posterior part of the palate (from the rugae up to the end of the soft palate). Four illustrations represent areas in the lower jaw: the lower lip, floor of the mouth, posterior part of the tongue (from vallate papilla up to end of the tongue), and anterior part of the tongue (from tip of the tongue up to vallate papilla). Finally, one illustration represents the pharynx. At each location, the patient can indicate the severity of the perceived oral dryness using a 5-point Likert scale ranging from 1 = “no dryness” to 5 = “severe dryness.”

#### Clinical oral dryness score

During the visit to the saliva clinic, the Clinical Oral Dryness Score (CODS) was scored for all patients by a single examiner (DHJJ). The CODS was recorded before determining the salivary flow rates and analyzing the xerostomia questionnaires, so the examiner was not aware during the recording of the CODS whether a patient suffered from hyposalivation/xerostomia or not.

The examiner scored the patient’s mouth for the presence or absence of ten features of oral dryness: (1) mirror sticks to buccal mucosa; (2) mirror sticks to tongue; (3) tongue shows loss of papillae; (4) tongue lobulated/fissured; (5) frothy saliva; (6) no saliva pooling in floor of mouth; (7) glassy appearance of other oral mucosa, especially palate; (8) debris on palate (excluding debris under dentures); (9) altered/smooth gingival architecture; and (10) active or recently restored (last 6 months) cervical caries (> 2 teeth) [27]. A specially designed form with illustrations of dry-mouth features from the original publication was used to score each feature [27]. The scores from the ten features were added together resulting in a total CODS ranging from 0 (no oral dryness) to 10 (extreme oral dryness).

#### Sialometry

The patients were instructed not to eat, drink, chew gum, brush teeth, use mouthwash, or smoke for at least 1 h before their visit to the saliva clinic. The procedure to determine the saliva secretion rate has been described by Jager and co-workers [28]. At the time of the collection of saliva, patients were placed in a quiet room and asked to sit in an upright position. The UWS was collected by the draining method in a pre-weighed plastic container [29]. To collect unstimulated saliva, patients were asked to immediately collect saliva after an initial swallow. Afterwards, they were asked to expectorate in the container as soon as they collected saliva. During saliva collection, the patients were not allowed to swallow. To collect CH-SWS, patients were asked to chew a 5 × 5-cm sheet of parafilm (Parafilm M, Pechiney, Chicago, IL, USA) with a frequency of approximately 60 chews per minute. The patients were instructed to expectorate the saliva every 30 s into a pre-weighed plastic container during a 5-min period. For stimulation of A-SWS secretion, a citric acid solution (2% *w*/*v*) was applied with cotton buds on the lateral borders of the tongue at 30-s intervals [30]. After the collection period was finished, the plastic containers were reweighted, and the collected volume was determined by subtracting the weight of the container prior to collection. The salivary flow was calculated by dividing the collected volume (assuming 1 g of saliva equals 1 mL) with collection time (min). Salivary flow rates were expressed in mL/min [29].

To determine whether patients suffered from hyposalivation, the following cut-off values were used: UWS < 0.10 mL/min, CH-SWS < 0.70 mL/min, and A-SWS < 0.70 mL/min [8].

### Data analysis

The data were processed in Microsoft Excel and then converted into SPSS, version 25.0 (IBM Corp SPSS Statistics, Armonk, NY, USA) for the statistical analysis. The Shapiro–Wilk test was used to assess the normality of the data. The data were presented as median, and their interquartile range (IQR) as all parameters were not normally distributed. The mean and standard deviation were also reported to clarify relatively small differences.

A Friedman test was conducted for the scores of the RODI and XI-scores, followed by a Wilcoxon signed-rank test as post hoc procedure.

Possible relationships among the RODI scores of the nine intra-oral regions, and the relation of the RODI scores with XI scores, UWS, CH-SWS, and A-SWS salivary flow rates were analyzed with a bootstrapped Spearman rank correlation test (1000 × bootstrapping). The Spearman’s rho coefficient and bias-corrected accelerated (Bca) 95% confidence interval were extracted. A significance level (*α*) of 0.01 was chosen for the correlation test.

The Mann-Whitney *U* test (significance level of *α* = 0.05) was performed to explore a possible relation between a positive CODS score and the associated region in the RODI.

## Results

A total of 337 patients participated in this study with an average age of 54 ± 17 years. The majority of the patients were female (68.5%). The RODI scores, XI-scores, CODS and UWS, CH-SWS, and A-SWS salivary flow rates were not normally distributed (Shapiro–Wilk test; *p* < 0.01). Table 1 presents the different salivary flow rates of the study population. Based on the UWS, CH-SWS, and A-SWS flow rates, respectively, 26.9%, 48.6%, and 13.1% of the study population suffered from hyposalivation.

**Table 1:** The unstimulated whole saliva (UWS), chewing-stimulated whole saliva (CH-SWS), and acid-stimulated whole saliva secretion rates of the study population. Data are expressed as the median with the corresponding interquartile range (IQR), and mean with standard deviation (SD)

	Median	IQR	Mean	SD	*N*
UWS (mL/min)	0.18	0.08–0.34	0.27	0.33	264
CH-SWS (mL/min)	0.70	0.34–1.18	0.89	0.84	313
A-SWS (mL/min)	1.80	1.05–2.78	2.00	1.23	321

### Regional Oral Dryness Inventory

In Table 2, the median and the corresponding IQR, and mean with standard deviation are shown for each of the nine intra-oral regions of the RODI. There was a significant difference in perceived oral dryness between the nine intra-oral regions (Friedman test *p* < 0.05, followed by Wilcoxon signed-rank tests *p* < 0.05). The highest scores were obtained for the posterior palate, while the lowest scores were obtained for the floor of the mouth (Table 2).

**Table 2:** Perceived oral dryness in nine different intra-oral regions as determined with the Regional Oral Dryness Inventory (RODI) in patients visiting a saliva clinic. Data are presented as median with corresponding interquartile range (IQR) and mean with standard deviation (SD)

	Median	IQR	Mean	SD	*N*
Upper lip	3.0	2.0–4.0	2.80	1.26	303
Anterior palate	3.0	1.0–4.0	2.82	1.40	302
Inside cheeks^a,b^	3.0	1.0–4.0	2.68	1.34	302
Posterior palate^a,b, c^	3.0	2.0–4.0	3.09	1.35	302
Lower lip^d^	3.0	2.0–4.0	2.70	1.26	299
Floor of the mouth^a,b,c,d,e^	2.0	1.0–4.0	2.54	1.34	297
Posterior tongue^a,b,c,e,f^	3.0	2.0–4.0	3.03	1.32	297
Anterior tongue^a,c,d,e,f^	3.0	2.0–4.0	2.94	1.40	297
Pharynx^a,b,c,d,e,f^	3.0	2.0–4.0	2.96	1.36	297

Wilcoxon signed-rank tests: ^a^*p* < 0.05 vs upper lip, ^b^*p* < 0.05 vs anterior palate, ^c^*p* < 0.05 vs inside cheeks, ^d^*p* < 0.05 vs posterior palate, ^e^*p* < 0.05 vs lower lip, ^f^*p* < 0.05 vs floor of mouth, ^g^*p* < 0.05 vs posterior tongue, and ^h^*p* < 0.05 vs anterior tongue

The scores of all regions correlate significantly with each other (Table 3) indicating that patients who suffer from severe xerostomia at one location in general also have high levels of xerostomia at other intra-oral locations. The correlation coefficient varies between 0.51 (pharynx with lower lip) and 0.82 (lower lip and upper lip). Four different regions have a correlation coefficient ≥ 0.75: the lower lip and upper lip, the posterior palate and posterior tongue, the anterior tongue and posterior tongue, and the floor of the mouth and inside the cheeks. The correlations of the scores between these four regions can be considered strong, whereas the other regions have a moderate correlation according to the standards described by Mukaka and co-workers and Akoglu and co-workers [31, 32].

**Table 3:** Correlation of the nine regions of the Regional Oral Dryness Inventory, *r:* Spearman’s rho correlation coefficient (BCa 95% confidence interval)

	Upper lip	Anterior palate	Inside Cheeks	Posterior palate	Lower lip	Floor of the mouth	Posterior tongue	Anterior tongue	Pharynx
Upper lip		r 0.69 (0.61–0.76)^*^	r 0.65 (0.56–0.72)^*^	r 0.56 (0.46–0.65)^*^	r 0.82 (0.76–0.86)^*^	r 0.61 (0.51–0.70)^*^	r 0.54 (0.44–0.63)^*^	r 0.57 (0.47–0.66)^*^	r 0.54 (0.44–0.63)^*^
Anterior palate			r 0.72 (0.65–0.78)^*^	r 0.73 (0.66–0.79)^*^	r 0.65 (0.57–0.72)^*^	r 0.70 (0.62–0.76)^*^	r 0.66 (0.58–0.75)^*^	r 0.67 (0.58–0.75)^*^	r 0.58 (0.49–0.67)^*^
Inside cheeks				r 0.65 (0.56–0.73)^*^	r 0.65 (0.56–0.72)^*^	r 0.75 (0.68–0.82)^*^	r 0.65 (0.56–0.74)^*^	r 0.64 (0.54–0.72)^*^	r 0.61 (0.51–0.70)^*^
Posterior palate					r 0.56 (0.47–0.64)^*^	r 0.69 (0.61–0.76)^*^	r 0.79 (0.73–0.85)^*^	r 0.65 (0.57–0.73)^*^	r 0.69 (0.61–0.76)^*^
Lower lip						r 0.67 (0.60–0.74)^*^	r 0.55 (0.45–0.63)^*^	r 0.63 (0.54–0.71)^*^	r 0.51 (0.41–0.59)^*^
Floor of mouth							r 0.73 (0.66–0.79)^*^	r 0.72 (0.66–0.78)^*^	r 0.65 (0.56–0.73)^*^
Posterior tongue								r 0.75 (0.67–0.81)^*^	r 0.71 (0.63–0.78)^*^
Anterior tongue									r 0.57 (0.47–0.65)^*^
Pharynx									

* = *p* < 0.01

The RODI scores at the nine intra-oral regions showed a weak to non-significant negative correlations with the UWS, CH-SWS, and A-SWS with Spearman’s rho correlation coefficient varying between − 0.27 and − 0.13.

### Xerostomia Inventory

Table 4 shows that the median of the 11 items of the XI varies between 2.0 and 4.0. There was a significant difference in perceived oral dryness and mouth feel between the 11 items of the XI (Friedman test *p* < 0.05, followed by Wilcoxon signed-rank tests *p* < 0.05). The XI item 4 (my mouth feels dry) had the highest scores and items XI 1 (sip liquids to swallow food) and XI 7 (I have difficulty swallowing food) had the lowest scores. The scores on the nine areas of the RODI correlate significantly with all items of the XI (data not shown) (presented in Appendix 1, for review purposes only). The highest correlation coefficient was observed between XI item 4 (mouth feel dry) and the dryness of the anterior tongue (*r* = 0.70). XI items related to extra-oral regions have poor correlations with RODI scores (correlation coefficient varying between 0.21 and 0.49) according to the standards described by Mukaka and co-workers and Akoglu and co-workers, for example, items 8 (skin of face), 9 (eyes), and 11 (nose) [31, 32]. In contrast, scores on XI item related to intra-oral locations show a stronger correlation with and the associated region of the RODI. Mainly XI item 7 (difficulty swallowing certain food) and XI item 10 (lips feel dry) have the highest correlation with the local dryness of respectively the pharynx (*r* = 0.56) and both upper and lower lip (*r* = 0.63 and 0.62).

**Table 4:** The scores of the 11 Xerostomia Inventory items (XI), presented as median with the corresponding interquartile range (IQR), and the mean with standard deviation (SD). *N* is the total numbers of participants

	Median	IQR	Mean	SD	*N*
XI 1 (sip liquids to swallow food)	2.0	1.0–4.0	2.61	1.59	336
XI 2 (mouth dry when eating a meal) ^a^	3.0	1.0–4.0	2.93	1.46	329
XI 3 (get up night to drink) ^a,b^	3.0	2.0–5.0	3.19	1.49	336
XI 4 (my mouth feels dry) ^a,b,c^	4.0	3.0–5.0	3.84	1.30	334
XI 5 (difficulty eating dry foods) ^a,d^	3.0	1.0–5.0	3.03	1.59	336
XI 6 (suck sweets to relieve dry mouth) ^b,c,d,e^	2.0	1.0–4.0	2.69	1.64	336
XI 7 (difficulty swallowing certain foods) ^b,c,d,e^	2.0	1.0–4.0	2.55	1.52	337
XI 8 (the skin of my face feels dry) ^a,c,d,e,g^	3.0	1.0–4.0	2.80	1.47	334
XI 9 (my eyes feel dry) ^a,d,f,g,h^	3.0	1.0–5.0	3.05	1.58	337
XI 10 (my lips feel dry) ^a,b,c,d,e,f,g,h,i^	4.0	3.0–5.0	3.63	1.34	337
XI 11 (the inside of my nose feels dry) ^a,c,d,f,g,j^	3.0	1.0–4.0	2.91	1.54	335
XI total	33.0	22.5–43.0	32.94	11.88	337

Wilcoxon signed-rank tests: ^a^*p* < 0.05 vs XI 1, ^b^*p* < 0.05 vs XI 2, ^c^*p* < 0.05 vs XI 3, ^d^*p* < 0.05 vs XI 4, ^e^*p* < 0.05 vs XI 5, ^f^*p* < 0.05 vs XI 6, ^g^*p* < 0.05 vs XI 7, ^h^*p* < 0.05 vs XI 8, ^i^*p* < 0.05 vs XI 9, ^j^*p* < 0.05 vs XI 10

### Clinical Oral Dryness Score

The median CODS of 319 persons is 4.0 with IQR of 2.0–5.0 (mean = 3.57, SD = 1.82).

Table 5 presents how frequently each item of the CODS was scored. In the overall study population, item 1 (the mirror sticks to the cheek; 78.9%) was most frequently scored, and item 8 (debris on the palate; 2.5%) the least. The presence of CODS item 1 (mirror sticks to buccal mucosa) was associated with a significant difference in dry-mouth feeling inside the cheeks (Mann-Whitney *U* = 4897, *p* = 0.009, *r* = − 0.16). CODS item 2 (mirror sticks to tongue) and CODS item 4 (tongue lobulated/fissured) were associated with higher dryness of the regions anterior and posterior tongue (CODS 2 respectively for anterior and posterior tongue; *U* = 6960, *p* = 0.000, *r* = − 0.26 and *U* = 7520, *p* = 0.000, *r* = − 0.21) (CODS 4 respectively anterior and posterior tongue; *U* = 5424, *p* = 0.000, *r* = − 0.22 and *U* = 6208, *p* = 0.023, *r* = − 0.14). CODS item 6 (no saliva pooling in floor of mouth) corresponds with a higher dry-mouth feeling of the floor of the mouth (*U* = 4466, *p* = 0.006, *r* = − 0.16). CODS item 7 (glassy appearance of oral mucosa especially palate) was associated with more severe oral dryness of the anterior and posterior palate (*U* = 7058, *p* = 0.000, *r* = − 0.27 and *U* = 6541, *p* = 0.000, *r* = − 0.31 respectively anterior and posterior palate). There were no significant relations between CODS item 3 (tongue shows loss of papillae) and item 8 (debris on palate and perceived oral dryness of the corresponding anatomical regions).

**Table 5:** Percentage of how frequently each item of the Clinical Oral Dryness Score (CODS) was identified (*N* = 319)

	CODS % yes
CODS 1 (mirror sticks to buccal mucosa)	78.9%
CODS 2 (mirror sticks to tongue)	48.7%
CODS 3 (tongue lobulated/fissured)	19.2%
CODS 4 (tongue shows loss of papillae)	24.8%
CODS 5 (frothy saliva)	61.8%
CODS 6 (no saliva pooling in floor of mouth)	19.2%
CODS 7 (glassy appearance of other oral mucosa especially palate)	47.4%
CODS 8 (debris on palate)	2.5%
CODS 9 (altered/smooth gingival architecture)	21.6%
CODS 10 (active or recently restored cervical caries)	36.4%

All the reported significant associations can be considered robust to distributional violations as the bootstrapped 95% confidence interval did not exceed 0.

## Discussion

The results of this study demonstrated intra-oral differences in perceived mouth dryness between different locations in the mouth by using the RODI, a recently developed xerostomia questionnaire. The perceived dryness was considered the highest for the posterior palate and the lowest for the floor of the mouth. The highest correlations were found between regions in the RODI and corresponding related individual items of the XI and CODS.

As described in the introduction, the saliva film on intra-oral tissue has local differences. The saliva film is thinnest at the anterior hard palate (~ 10 μm), while the saliva film at the anterior dorsal area of the tongue is much thicker (~ 54 μm) [18]. This pattern of different saliva film thickness at various intra-oral locations has been confirmed by other studies, where the palate is considered most dry, and tongue and floor of the mouth are considered as most wet, which explains the high MUC5 concentration on the palate [17, 19–21].

Several factors make the hard palate more susceptible to oral dryness compared with other intra-oral locations; paucity of (hard) palatal glands, gravity, and evaporation [1, 19, 33]. Gravity forces part of the whole saliva to pool in the floor of the mouth between swallowing episodes. As a consequence, the palate can be insufficiently moistened, especially in case of hyposalivation [20]. Furthermore, the palate is more prone to saliva evaporation, especially during speaking and breathing; and during speech air passes more or less continuously from the lungs over the mucosa of the palate [19]. The advantage of the tongue is that it is located near the opening from Wharton’s ducts [17, 19, 20]. Here, saliva from the many minor glands in this region and the nasopalatine glands as well as the secretions of the submandibular and sublingual glands is collected [20]. This pattern of saliva thickness on the various mucosal sites does not only apply to healthy subjects but is also applicable for dry-mouth patients [18, 20, 21].

The current study found intra-oral differences in perceived mouth dryness, in line with previous research finding different saliva film thickness at different intra-oral locations. This present study found that the posterior palate was experienced as most dry, whereas other studies indicated that the anterior hard palate had the thinnest saliva coating [17, 18, 21]. The latter region is comparable with the anterior palate in this study. A possible explanation for this difference could be that patients find it hard to distinguish between two directly adjacent regions: the anterior part (up to the rugae) and posterior part (from the rugae to the end of the soft palate) of the palate and the posterior palate and the pharynx. In both cases, these regions have higher correlations compared with that of non-adjacent regions.

Another study reported the whole hard palate as having the thinnest saliva film without making a distinction between the anterior and posterior part [19]. Our results are in line with this study, as the schematic illustration of the posterior palate in the RODI is a combination of the hard palate and soft palate, which partly resembles the area studied by DiSabato-Mordarski and co-workers. Wolff and co-workers concluded that mostly hyposalivation patients had lower saliva film thickness at the posterior palate about 5-mm palatal to the second molars [20]. This could indicate that these patients could experience more dryness at the soft palate which is a part of the posterior palate in the present study.

In our study, the floor of the mouth was the wettest of all intra-oral regions. This finding is in line with previous studies [19, 20]. Another study also showed that the CODS item, no saliva pooling in the floor of the mouth, was only scored positively in the most severe hyposalivation patients [28]. However, three other studies only indicated the dorsal surface of the tongue as most wet [17, 18, 21]. These differences can be explained by the fact that these studies only measured the saliva thickness at the tongue and did not investigate the floor of the mouth.

The salivary flow rates had only negligible correlations with the perceived oral dryness at the nine regions. This supports the hypothesis that flow rates and severity of xerostomia do not have to be correlated [16, 23, 34]. Pai and co-workers explored self-reported dryness at four locations (lips, mouth, tongue, and throat) with a Visual Analogue Scale (VAS). They also found that the VAS scores showed little or no significant correlations with salivary flow rates [35].

Although the XI has been developed to quantify the overall feeling of mouth dryness, it contains some items referring to the dryness at different parts of the body, for example the lips, the eyes, the skin, and the inside parts of the nose. As expected, XI items on extra-oral regions had poor correlations with regions of the RODI, whereas XI items related to dryness of the lips and difficulty in swallowing correlated higher with respectively upper and lower lip and pharynx of the RODI compared with all other regions. The regionally related CODS items also had a significant association with related regions in the RODI.

This study has some potential limitations. The patients who participated in this study are patients referred to a specialized saliva clinic. These patients suffer from saliva-related complaints and might be more concerned about their oral dryness than average patients suffering from dry mouth. Therefore, the results of this study could not be extrapolated to healthy subjects and other patients with dry-mouth complaints yet, and further studies with the RODI in other groups of patients seem warranted.

These subsequent studies could also explore different groups of patients, grouped according to the etiological factors for oral dryness. It is feasible that patients with oral dryness due to irradiation of the head and/or neck region might have another pattern of intra-oral dryness than patients suffering from Sjögren’s disease or medication-induced hyposalivation.

## Main conclusions

The present study suggests that there is a significant difference in dry-mouth feeling among different intra-oral locations, with the highest perceived oral dryness for the posterior palate and the lowest for the floor of the mouth. Introduction of the RODI might help to discriminate among different potential causes of oral dryness in patients and for evaluating the efficacy of mouth-moistening products.

## Electronic supplementary material

ESM 1 (DOCX 16 kb)
