# Supplementary material for: Regional differences in perceived oral dryness as determined with a newly developed questionnaire, the Regional Oral Dryness Inventory
Source: Clin Oral Investig. 2020 May 7;24(11):4051–60. doi: 10.1007/s00784-020-03276-7 (PMC7544722; doi:10.1007/s00784-020-03276-7)
Supplement: Supplementary file 1 — (DOCX 16 kb) [file 784_2020_3276_MOESM1_ESM.docx]

|  | **XI 1** | **XI 2** | **XI 3** | **XI 4** | **XI 5** | **XI 6** | **XI 7** | **XI 8** | **XI 9** | **XI 10** | **XI 11** | **XI tot** |
| --- | --- | --- | --- | --- | --- | --- | --- | --- | --- | --- | --- | --- |
| **Upper lip** | r 0.46  (0.36- 0.56)^*^ | r 0.56  (0.47-0.63)^*^ | r 0.42  (0.32- 0.51)^*^ | r 0.57  (0.49-0.65)^*^ | r 0.52  (0.42-0.60)^*^ | r 0.38  (0.27-0.48)^*^ | r 0.47  (0.37-0.55)^*^ | r 0.30  (0.18-0.41)^*^ | r 0.36  (0.25-0.47)^*^ | r 0.63 (0.54- 0.71)* | r 0.40  (0.30-0.50)* | r 0.62  0.54-0.69)* |
| **Anterior palate** | r 0.40  (0.29-0.51)^*^ | r 0.48  (0.38-0.58)^*^ | r 0.42  (0.32-0.50)^*^ | r 0.63  (0.55-0.71)^*^ | r 0.48  (0.38-0.59)^*^ | r 0.39  (0.28-0.49)^*^ | r 0.44  (0.35-0.54)^*^ | r 0.21  (0.10-0.33)^*^ | r 0.28  (0.17-0.39)^*^ | r 0.47  (0.38-0.57)* | r 0.35 (0.24-0.45)* | r 0.56 (0.48-0.65)^*^ |
| **Inside cheeks** | r 0.41  (0.30-0.52)^*^ | r 0.54  (0.44-0.63)^*^ | r 0.40  (0.29-0.49)^*^ | r 0.64 (0.56-0.72)^*^ | r 0.49  (0.40-0.58)^*^ | r 0.39  (0.28-0.49)^*^ | r 0.48  (0.39-0.56)^*^ | r 0.24  (0.13-0.36)^*^ | r 0.30  (0.18-0.42)^*^ | r 0.51  (0.42-0.60)^*^ | r 0.46 (0.35-0.56)^*^ | r 0.60 (0.52-0.67)^*^ |
| **Posterior palate** | r 0.42  (0.32-0.52)^*^ | r 0.51  (0.39-0.62)^*^ | r 0.49  (0.38-0.58)^*^ | r 0.69  (0.61-0.75)^*^ | r 0.49  (0.39-0.59)^*^ | r 0.43  (0.33-0.53)^*^ | r 0.47  (0.37-0.56)^*^ | r 0.30  (0.18-0.41)^*^ | r 0.37  (0.27-0.48)^*^ | r 0.43  (0.33-0.53)^*^ | r 0.40 (0.29-0.51)^*^ | r 0.62  (0.54-0.70)^*^ |
| **Lower lip** | r 0.49  (0.39-0.57)^*^ | r 0.54  (0.44-0.61)^*^ | r 0.34  (0.23-0.44)^*^ | r 0.57  (0.49-0.65)^*^ | r 0.52  (0.43-0.60)^*^ | r 0.38  (0.28-0.48)^*^ | r 0.48  (0.39-0.57)^*^ | r 0.34  (0.23-0.44)^*^ | r 0.36  (0.25-0.47)^*^ | r 0.62  (0.53-0.71)^*^ | r 0.40  (0.30-0.50)^*^ | r 0.62  (0.54-0.68)^*^ |
| **Floor of mouth** | r 0.40  (0.29-0.52)^*^ | r 0.53  (0.44-0.62)^*^ | r 0.38  (0.28-0.48)^*^ | r 0.63  (0.56-0.70)^*^ | r 0.50  (0.40-0.60)^*^ | r 0.44  (0.34-0.54)^*^ | r 0.44  (0.35-0.54)^*^ | r 0.27  (0.15-0.39)^*^ | r 0.31  (0.20-0.42)^*^ | r 0.47  (0.36-0.57)^*^ | r 0.40  (0.29-0.51)^*^ | r 0.58  (0.50-0.66)^*^ |
| **Posterior tongue** | r 0.39  (0.29-0.50)^*^ | r 0.54  (0.44-0.64)^*^ | r 0.41  (0.32-0.51)^*^ | r 0.68  (0.61-0.75)^*^ | r 0.52  (0.42-0.61)^*^ | r 0.46  (0.36-0.55)^*^ | r 0.50  (0.40-0.58)^*^ | r 0.30  (0.18-0.42)^*^ | r 0.31  (0.20-0.43)^*^ | r 0.45  (0.35-0.54)^*^ | r 0.41  (0.30-0.52)^*^ | r 0.59  (0.52-0.67)^*^ |
| **Anterior tongue** | r 0.40  (0.29-0.51)^*^ | r 0.56  (0.47-0.64)^*^ | r 0.40  (0.30-0.50)^*^ | r 0.70  (0.63-0.76)^*^ | r 0.55  (0.45-0.64)^*^ | r 0.54  (0.45-0.62)^*^ | r 0.50  (0.40-0.59)^*^ | r 0.27  (0.16-0.38)^*^ | r 0.29  (0.17-0.41)^*^ | r 0.49  (0.37-0.59)^*^ | r 0.36 (0.24-0.48)^*^ | r 0.61  (0.52-0.68)^*^ |
| **Pharnyx** | r 0.46  (0.36-0.57)^*^ | r 0.55  (0.45-0.64)^*^ | r 0.42  (0.31-0.53)^*^ | r 0.57  (0.48-0.65)^*^ | r 0.53  (0.44-0.61)^*^ | r 0.32  (0.21-0.44)^*^ | r 0.56  (0.47-0.64)^*^ | r 0.37  (0.25-0.49)^*^ | r 0.41  (0.31-0.52)^*^ | r 0.42  (0.31-0.52)^*^ | r 0.49  (0.40-0.59)^*^ | r 0.63  (0.55-0.71)^*^ |

Appendix 1

Table: Correlation of the Xerostomia Inventory (XI) total scores with the scores of perceived oral dryness in the nine different intra-oral regions, r: Spearman’s rho correlation coefficient (BCa 95% confidence interval).

*p< 0.01
